# Paper-Based Supercapacitive Mechanical Sensors

**DOI:** 10.1038/s41598-018-34606-1

**Published:** 2018-11-02

**Authors:** Ye Zhang, Serdar Sezen, Mahdi Ahmadi, Xiang Cheng, Rajesh Rajamani

**Affiliations:** 1 Department of Mechanical Engineering, University of Minnesota, 111 Church St. SE, Minneapolis, MN 55455 USA; 2 Department of Mechanical and Manufacturing Engineering, St. Cloud State University, 720 Fourth Avenue South, Saint Cloud, MN 56301 USA; 3 Department of Chemical Engineering and Materials Science, University of Minnesota, 421 Washington Ave. SE, Minneapolis, MN 55455 USA

## Abstract

Paper has been pursued as an interesting substrate material for sensors in applications such as microfluidics, bio-sensing of analytes and printed microelectronics. It offers advantages of being inexpensive, lightweight, environmentally friendly and easy to use. However, currently available paper-based mechanical sensors suffer from inadequate range and accuracy. Here, using the principle of supercapacitive sensing, we fabricate force sensors from paper with ultra-high sensitivity and unprecedented configurability. The high sensitivity comes from the sensitive dependence of a supercapacitor’s response on the contact area between a deformable electrolyte and a pair of electrodes. As a key component, we develop highly deformable electrolytes by coating ionic gel on paper substrates which can be cut and shaped into complex three-dimensional geometries. Paper dissolves in the ionic gel after determining the shape of the electrolytes, leaving behind transparent electrolytes with micro-structured fissures responsible for their high deformability. Exploiting this simple paper-based fabrication process, we construct diverse sensors of different configurations that can measure not just force but also its normal and shear components. The new sensors have range and sensitivity several orders of magnitude higher than traditional MEMS capacitive sensors, in spite of their being easily fabricated from paper with no cleanroom facilities.

## Introduction

Paper offers multiple advantages as a substrate material for sensors: it is ubiquitous, low-cost, lightweight and biodegradable^1^. Paper-based sensors would be especially valuable for health care diagnostics in developing countries and for other similar cost sensitive applications^2^. Recently, researchers have developed paper-based sensors, which could provide highly inexpensive microfluidic devices^3,4^ and portable bio-assays^5–7^ for detecting analytes in point of care health diagnostics. Devices fabricated from paper also include respiration analysis sensors^8^, textile sensors^9^, capacitive touch sensors^10^ and microelectronics^2,11^. Paper is porous and made up of a network of cellulose fibers, which offers unique structural properties. It is naturally hydrophilic and readily absorbs aqueous liquids that spread inside the paper through capillary flow within its fiber matrix^12^. The cellulose fibers can also be functionalized, thus tuning their properties such as hydrophilicity, if desired, as well as their permeability and reactivity^13^. As a result, fabrication methods for paper-based sensors are extremely flexible, ranging from cutting, wax printing, screen printing, and analogue plotting to photolithography, inkjet printing and etching, plasma treatment, flexography printing, and laser treatment^12,14–16^. Although tremendous progress has been made on paper-based sensors, the use of paper as a substrate for mechanical sensors is in its preliminary stages. Some pioneering works in this area include piezoresistive force sensors based on a carbon resistor patterned on paper^17^ and resistive strain gauges on paper^18,19^. Although these sensors can be very quickly fabricated and are highly inexpensive, their range of measurement is typically small, extending only up to 16 mN for force sensors^17^. These metrics compare poorly with the large range of traditional load cells and even with that of microelectromechanical system (MEMS) sensors.

**Figure 1:**
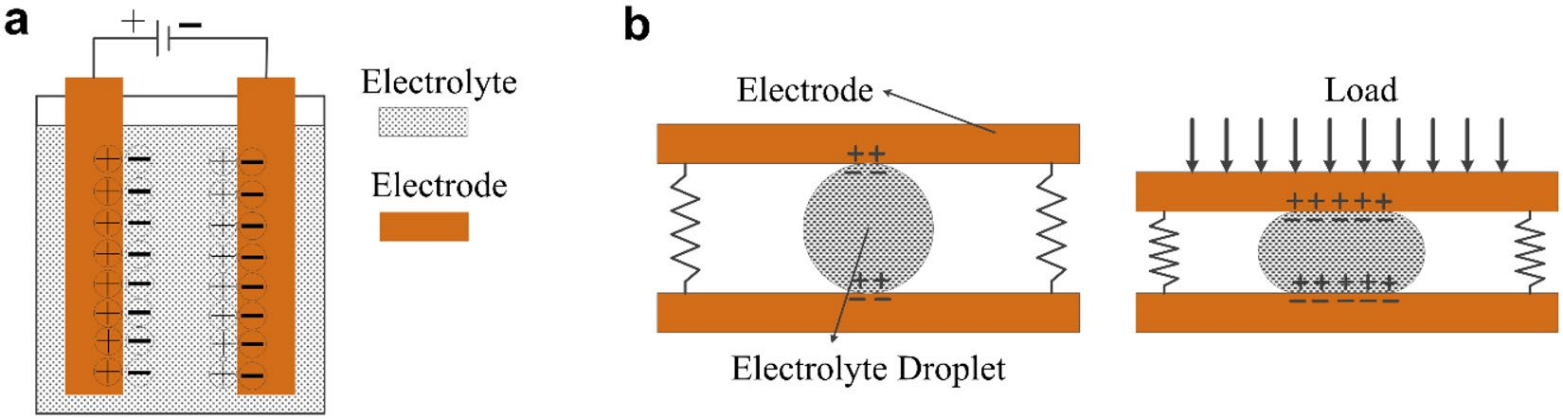
Supercapacitor and electrolyte-droplet-based supercapacitive force sensor. (**a**) Schematic of a supercapacitor. (**b**) Supercapacitor-based force sensor without load (Left) and with load (Right).

To overcome this limitation, we present a new fabrication method for making paper-based force sensors that outperform MEMS sensors on both sensitivity and range. The new paper-based sensors retain their low-cost advantage and can be quickly fabricated in a matter of minutes with no need for cleanroom facilities. Hence, these new sensors can have broad applications in several industrial and bio-medical contexts. The large range and high sensitivity of our paper-based sensors arise from the principle of supercapacitive sensing. Supercapacitors, including electrical double-layer capacitors (EDLCs), pseudocapacitors (PC) and hybrid supercapacitors have been widely studied for energy storage^20–31^. In this paper, only one type of supercapacitors, the EDLC, is discussed. The name “supercapacitor” is used to refer to all EDLCs in this paper. While a traditional capacitor is composed of parallel plate electrodes with a dielectric in between them, a supercapacitor consists of a pair of electrodes with an electrolyte solution in between. A basic form of an EDLC supercapacitor is shown in the schematic in Fig. 1a. The application of a voltage across the electrodes enables the flow of ionic current in the electrolyte. The positive and negative ions in the electrolyte separate from each other, forming positive and negative layers of charges at the two electrodes, respectively. The electrode-electrolyte interface at each electrode is therefore a double layer formed between the electrolyte ions and the electrode charges. The capacitance of a traditional parallel plate capacitive sensor is described by the well-known equation

1 $$C=\frac{\varepsilon A}{d}$$

where *A* is the geometric surface area of the electrode; *ε* is the relative permittivity of the dielectric material; and *d* is the distance between two oppositely biased electrodes. In contrast, in a supercapacitor, *A* is the effective area of the electrodes in contact with the electrolyte, which is typically high due to a selection of electrode materials which have high specific area and porosity. The variable *d* is determined by the double layer of atomic thickness at each electrode, and is of the order of Angstroms. Therefore, supercapacitors have significantly higher capacitances compared to traditional capacitors.

In a traditional capacitor-based force sensor, the distance *d* between electrodes changes due to applied force^32,33^, which results in a change in capacitance. Thus, the measurement of capacitance provides a measure of the force exerted. In a supercapacitive sensor, on the other hand, the distance *d* is fixed at the atomic double layer thickness. Instead, the area *A* changes in response to force. In currently-available supercapacitive force sensors^34–37^, the electrolyte is a liquid and the contact area *A* between this liquid and the electrodes changes by the application of a force. Figure 1b shows an example of a supercapacitive sensor in which a drop of electrolytic fluid is squeezed between two electrodes^37^. A force on the electrodes causes the drop to be squeezed, resulting in a change of *A*. This leads to a change in capacitance, which serves as a measure of the applied force. The surface of the electrodes is usually treated to be superhydrophobic, ensuring that the electrolyte does not “stick” to the electrodes and moves quickly in response to applied force without hysteresis. However, such liquid-state supercapacitive sensors have several inherent disadvantages, which limit the broad use of these sensitive sensors: (I) The sensor cannot be easily miniaturized to create micro-sensors, since it is difficult to create size-controlled micron-sized droplets or liquid pool and to trap the liquid inside a sealed sensor. (II) The presence of the hydrophobic coating on the surface of the electrodes reduces the capacitance, because it increases the distance between the electrolyte and the electrode. (III) Even in large-sized sensors, each sensor needs to be individually calibrated to account for variability in liquid size and location inside the sensor chamber. (IV) The high cost of the hydrophobic coating and the large size of the sensor pose problems in creating a sheet of such sensors for measuring distributed forces (e.g. forces from the foot of a patient as he/she walks). (V) The shelf life of liquid-state sensors is short due to atmospheric evaporation. (VI) The effect of gravity on the liquid may limit their application in systems involving non-planar motion.

In this manuscript, we use functionalized paper as a substrate for making highly flexible *solid*-state electrolytes in supercapacitive force sensors, which help address all of the above limitations of current supercapacitive sensors. The new electrolytes are made by introducing active materials into the porous matrix of paper and functionalizing the entire thickness of the paper. The deformation of the electrolytes in response to an applied force and the resulting change in its contact area with the electrodes can then be used to sense the applied force. In addition to the electrolyte, the entire sensor can also be made from paper substrates resulting in a simple and quick fabrication process.

A highly flexible solid-state electrolyte with a significant capability for deformation is essential for our paper-based force sensors. The new electrolyte is fabricated by mixing an ionic liquid with a photo-curable polymer, brushing this ionic mixture on to filter paper, and then exposing the combination to UV light for a short time duration (1 minute). Ionic liquids are good solvents for cellulose^38,39^. When the ionic gel mixture is coated on paper, it takes the shape of the filter paper, first filling the porous paper matrix and then slowly dissolving the paper over a period of approximately 25 minutes. Since the curing time for the ionic gel in the paper substrate (1 minute) is much less than the time taken for the paper to dissolve (25 minutes), the original shape of the paper substrate is retained by the ionic gel. The paper itself eventually dissolves, leaving a clear transparent and flexible ionic gel electrolyte. Because the original paper substrate can be cut, rolled, folded and shaped into various three-dimensional (3D) geometries, the electrolyte can also be easily made into different complex 3D shapes. The high flexibility of the new electrolyte, as shown in the next section, arises from the mechanical properties of the microstructure created by dissolution of the cellulose structure of the filter paper in the gel matrix. This highly flexible electrolyte makes possible the development of supercapacitive sensors, which incorporate significant bending or deformation as a part of the sensing mechanism.

## Results and Discussion

**Figure 2:**
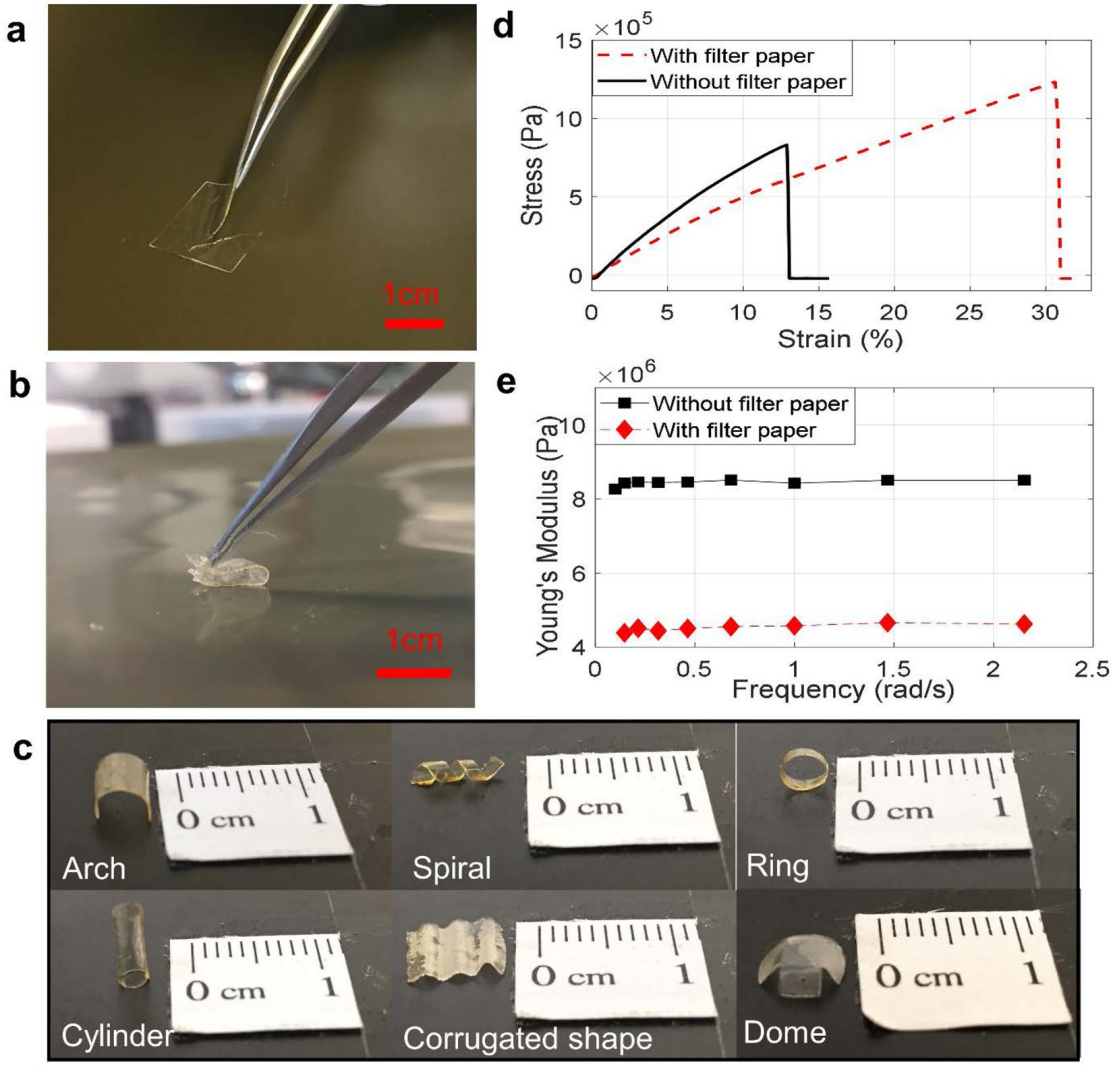
Flexibility of paper-based-electrolytes and their mechanical properties. (**a**) Failure of an ionic gel electrolyte without filter paper under bending. (**b**) Significantly higher flexibility of a paper-based electrolyte. (**c**) Various shapes of paper-based electrolytes. (**d**) Stress-strain curves of an ionic gel electrolyte and a paper-based electrolyte. (**e**) Young’s modulus of an ionic gel electrolyte and a paper-based electrolyte under tensile cyclic loads at low frequencies.

Ionic-gel electrolytes that incorporate ionic liquid into a cross-linkable polymer matrix provide both mechanical stability and high electrical conductivity^40–45^. However, a traditional ionic gel has very limited flexibility, and can only be utilized in the form of a thin flat film. Figure 2a shows the limited flexibility of an ionic-gel electrolyte, where a thin film of the solid-state ionic-gel electrolyte cracks while it is being bent. To use a solid-state electrolyte for supercapacitive sensing, good mechanical properties, ease of fabrication with desirable shapes, the ability to form good electrode/electrolyte contact, and high ionic conductivity are essential characteristics. By brushing the ionic gel on to filter paper before cross-linking, all of these properties can be obtained. Figure 2b shows the high flexibility of the paper-based electrolyte, where a thin film of the electrolyte can be bent and completely folded without mechanical failures.

Due to the softness and flexibility of the paper-based electrolyte, it can be easily shaped into complex geometries, which is hard, if not impossible, to achieve with the original ionic-gel electrolyte. Figure 2c shows some examples of electrolyte geometries created from the paper-based electrolytes, including a hollow cylinder, an arch, a ring, a corrugated element, a dome and a spiral. These geometries result in electrolyte structures with ultra-low stiffness, which is favorable when incorporated in supercapacitive sensors. Such supercapacitive sensors can deform significantly by force with dramatically increasing contact areas *A*.

### Properties of paper-based electrolytes

Thin films of ionic-gel electrolytes and paper-based electrolytes were stretched under tensile forces using dynamic mechanical analysis (DMA) and their stress-strain curves were obtained, as shown in Fig. 2d. In comparison with the ionic-gel electrolyte, the ultimate tensile strength (maximum stress before failure) of the paper-based electrolyte is 50% larger, the toughness 3.55 times higher and the maximum elongation strain 2.5 times larger. To further characterize the flexibility of the two electrolytes, Fig. 2e compares the Young’s modulus of the ionic-gel and paper-based electrolytes under cyclic tensile loads at low frequencies. The Young’s modulus of the paper-based electrolyte is only about half of the Young’s modulus of the ionic-gel electrolyte.

**Figure 3:**
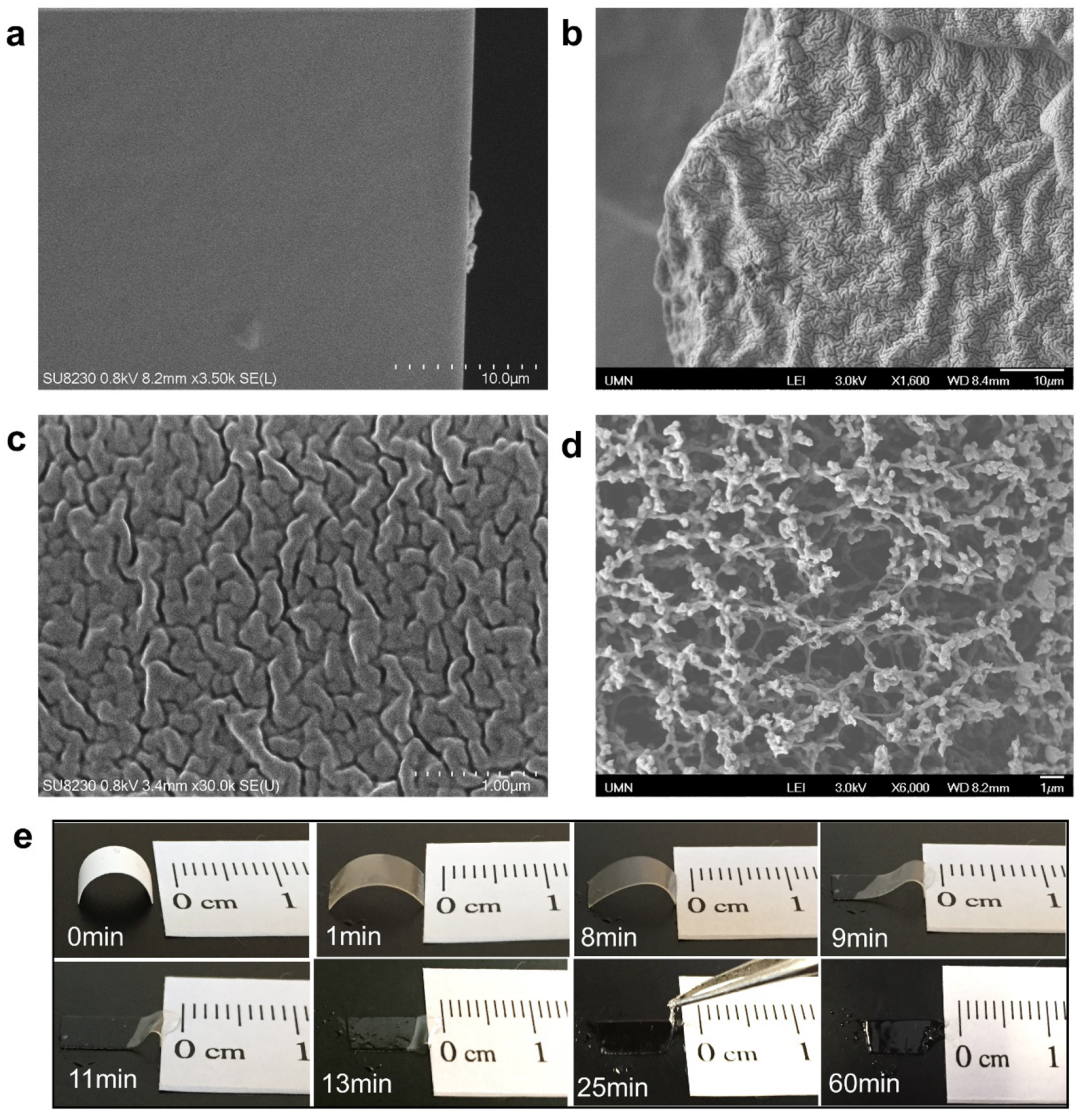
Microscopic structures of paper-based electrolytes. **(a)** SEM image of the cross-section of an ionic-gel electrolyte. (**b**) SEM image of the cross-section of a paper-based electrolyte. **(c)** Zoom-in view of the micron-sized wrinkles of the paper-based electrolyte. **(d)** SEM image of the filter paper. **(e)** Photographs showing the dissolving of filter paper in ionic gel. Cross-linking does not occur in this test.

The flexibility of the paper-based electrolytes comes from micro-wrinkles created by the slow dissolving in ionic gel of filter paper fibers. Scanning Electron Microscope (SEM) images of the cross-sections of the paper-based electrolytes show micron-sized fissures, creating a network with many wrinkles (Fig. 3b,c). In comparison, these fissures and wrinkles cannot be found in the cross-section of the ionic-gel electrolytes without filter paper (Fig. 3a). The fissures are vestiges of the fiber structures of the filter paper. The bulk of the material consists of PEG diacrylate (PEGDA), the cross-linked polymer with ionic gel. But the portions that contained cellulose fibers are filled with a softer material. Cellulose fibers get dissolved by the ionic gel into low molecular weight compounds^46,47^. A strong hydrogen-bonding interaction occurs between PEGDA and the dissolved cellulose molecules^48^. This weakens the crystallinity of PEGDA resulting in an improvement in the softness and extensibility properties of the film^48,49^. In short, the bulk of the material is the more-brittle PEGDA while the fissures are soft due to dissolving of the cellulose fibers in those locations. The fissures or wrinkles allow for greater compressibility and provide lower stiffness. Figure 3c shows a zoomed-in view of the wrinkles in the paper-based electrolytes. The size of the fissures is about 100–200 nm, matching well with the characteristic size of the cellulose fiber structure in the filter paper (Fig. 3d).

When brushing the ionic gel onto the filter paper and exposing the filter paper to UV, two processes happen: the crosslinking of the polymer network and the dissolution of the fiber structure of the filter paper. The dissolution process takes significantly longer time than the crosslinking process, so the shape of electrolytes determined by the initial filter paper substrate remains. The crosslinking of the polymer occurs on a barely dissolved structure and fills in the porous holes of the fiber structure of the filter paper. Therefore, the ionic gel takes a similar structural shape as that of the filter paper. The cellulose fibers in the filter paper eventually dissolve after a sufficiently long time, leaving the submicron fissures of the wrinkle structure shown in Fig. 3b,c. Thus, the key for the formation of the wrinkle structure is the separation of the time scales of the cross-linking and dissolution processes. We directly measured the dissolution time of filter paper in ionic gel without cross-linking (Fig. 3e). Right after brushing the gel onto the filter paper it starts turning transparent, but the shape of the filter paper remains. The filter paper dissolves slowly. At around 9 minutes after applying the gel, the electrolyte arch starts to collapse. After about 25 minutes, the arch shape collapses. Nevertheless, the edge of the paper stays. After 1 hour, the filter paper totally dissolves in the gel, loses its original shape and becomes a pool of gel without a definite shape. Hence, the dissolution time is more than 25 minutes, but less than 60 minutes. In contrast, the cross-liking under UV exposure takes 1 minute. The separation of these two-time scales explains the two key features of the paper-based electrolytes, i.e., the retaining of the electrolyte shape from the paper substrate and the creation of micron-sized fissures that leads to the high flexibility.

### Examples of paper-based supercapacitive force sensors

The new flexible paper-based electrolytes enable rapid fabrication of many different configurations of supercapacitive force sensors. The fabrication process is easy, inexpensive and highly adaptable to various applications. Four sensor embodiments using different geometries of paper-based electrolytes are presented below to illustrate the versatility of our sensors.

**Figure 4:**
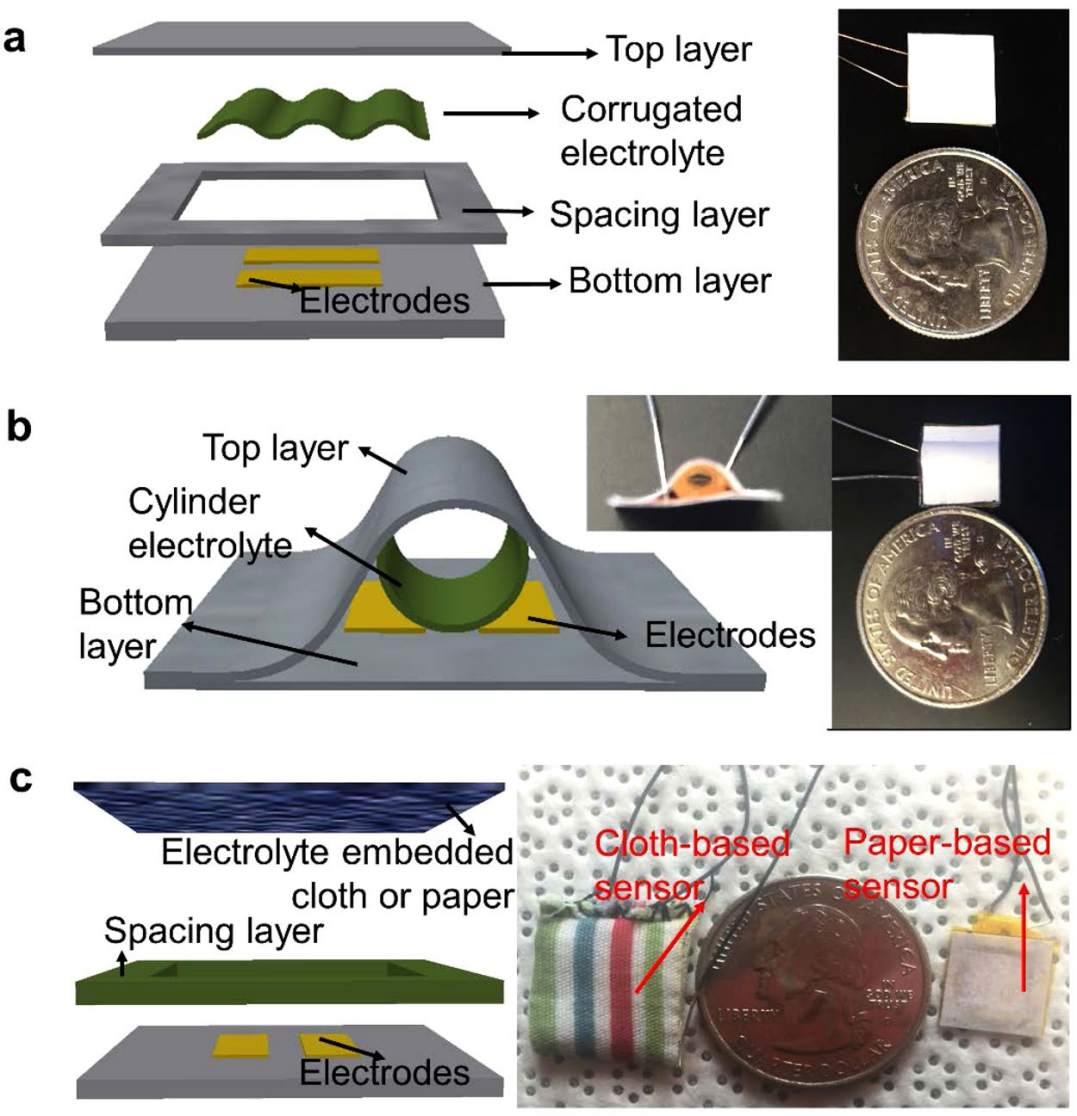
Embodiments of paper-based supercapacitive force sensors. (**a**) Schematic (left) and photograph (right) of a force sensor made of a corrugated electrolyte. (**b**) Schematic (left) and photograph (right) of a force sensor made of a hollow cylindrical electrolyte. (**c**) Schematic (left) and photograph (right) of force sensors made with cloth-based and paper-based electrolytes.

Figure 4a shows a prototype force sensor with a corrugated electrolyte element. In addition to its use in fabrication of the electrolyte, paper is also used as the structural material for both the top and bottom panels and the spacing layer. Two pieces of copper tape (0.06 mm thick) were cut and adhered to the bottom paper panel, as two electrodes. A spacing layer was glued to the bottom panel, with the electrodes exposed. The corrugated paper-based electrolyte was put on top of the electrodes before gluing the top panel to the spacing layer. When a load is applied on the top layer, the corrugated electrolyte deforms, resulting in an increase in the contact area of the valley of the corrugated electrolyte with the bottom electrodes, thus causing an increase in capacitance.

Figure 4b shows a prototype force sensor using an elastic paper-based rolled up electrolyte. The sensor consists of a planar set of two copper electrodes on a paper substrate and a rolled up hollow cylindrical electrolyte placed on top of the electrode pair. The copper electrodes were made by simply using adhesive copper tape and fixing them on the bottom sheet of paper. The two pieces of paper are glued together with the top layer bent into an arch shape. Due to the cylindrical electrolyte being hollow, it deforms easily under force and the contact area increases with the shape changing from circular to elliptical. The areal change is then translated into a capacitance change, which can be measured.

Figure 4c shows prototypes of cloth-based and regular-paper-based sensors. The supercapacitive sensors can be fabricated not only using filter paper, but also by using regular printing paper and even cloth. With regular paper and cloth, the dissolution of the fiber structure happens only to a partial degree. Figure 4c shows two prototypes of the supercapacitive force sensors, in which the ionic gel is brushed onto regular paper and cloth. The paper and cloth are partially dissolved, and the rest of the substrate structure holds the ionic gel, which can then be used as the electrolyte of supercapacitive force sensors. The sensors are made simply by sewing or gluing together two sheets of cloth/paper. The electrolyte is then brushed onto the top cloth/paper sheet and cured under UV light. The two parallel copper tape electrodes are on the bottom sheet of cloth/paper. For the cloth sensor, a frame made of carton was used as the spacing layer, while for the paper sensor, the spacing layer is made of regular paper. The top cloth/paper sheet behaves like a membrane with embedded electrolytes under the boundary condition of four fixed edges. When load is applied on the top layer, it deforms and contacts with the electrodes on the bottom layer, resulting in a change of the capacitance measured.

**Figure 5:**
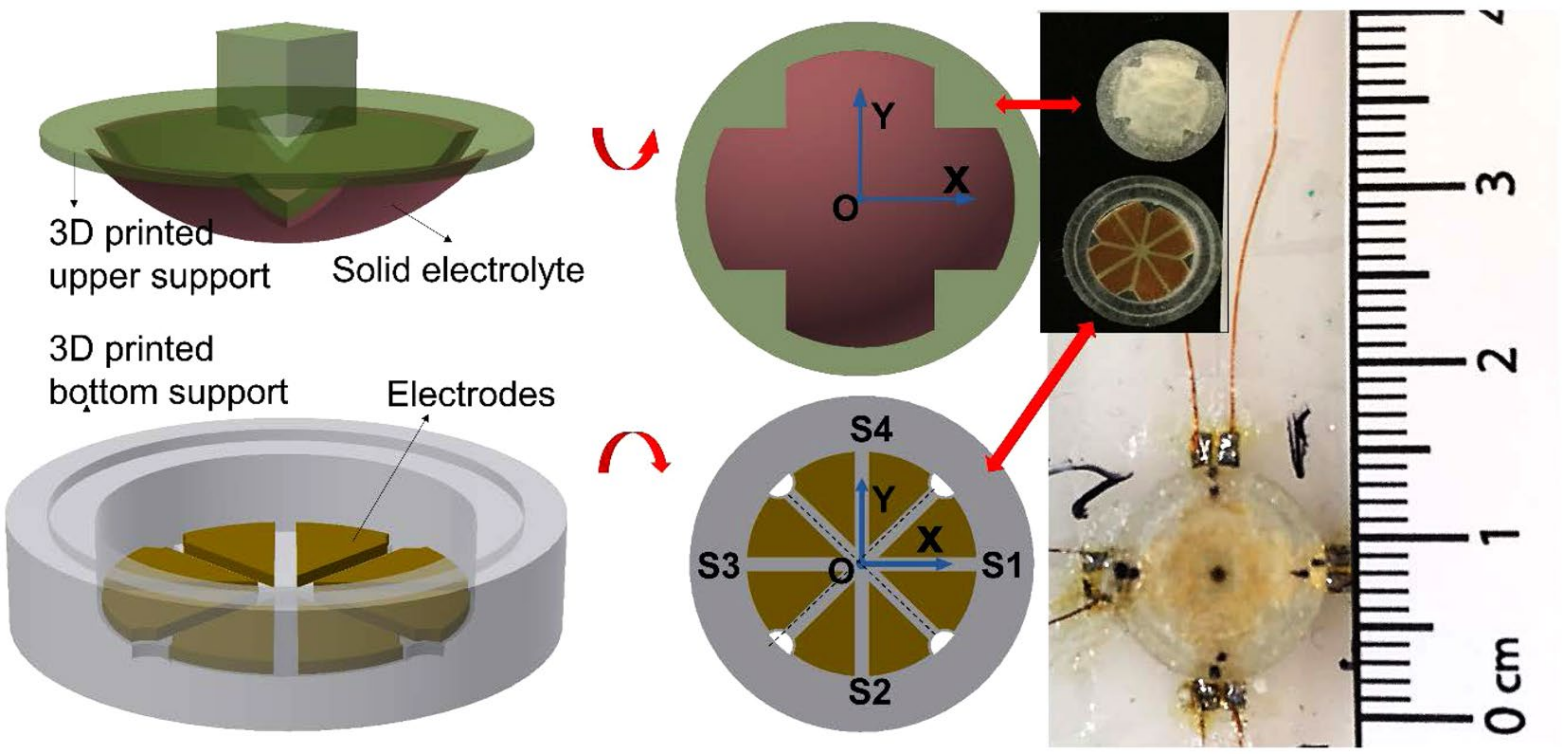
Embodiment of supercapacitive sensors for a more complicated force measurement: Force sensor array with a quad architecture for simultaneous measurement of normal and shear forces.

Figure 5 shows a prototype combined normal and shear force sensor. Due to the high sensitivity of supercapacitive sensing, an array of sensors can be integrated in a small area to obtain sophisticated combinations of measurements. For example, a quad-unit force sensing cell utilizing the paper-based electrolyte was fabricated, which simultaneously measures both normal and shear forces (Fig. 5). The cell consists of four separate force sensors. Each sensor has two metal electrodes. The electrodes are patterned on a flexible polyimide substrate in such a way that the four sensors lie symmetrically on the axes of a Cartesian coordinate system with the center of the eight electrodes located at the origin. The electrodes on the substrate were then assembled inside a 3D printed bottom support. The filter paper is pressed into a mold, which is transferred on the top of a 3D printed sphere surface. It is then brushed with the ionic gel and exposed to UV light for curing. The 3D printed sphere is then transferred and assembled on the bottom support using sealing glue. The 3D printed sphere is made of a soft elastic material (Agilusclear, Stratasys). When force is applied from the top, the sphere will deform, along with the electrolyte on the surface, resulting in a change of contact area between the electrolyte and four pairs of electrodes. The sensor is calibrated and calibration coefficients between force and capacitance of each sensor are obtained. The four capacitance readings (C1, C2, C3, C4) of the four sensors (S1, S2, S3, S4) are used to estimate the normal force and shear force applied on the sensing cell.

The normal force is obtained from the average of the four force sensors as

2 $${F}_{n}=\frac{1}{4}({K}_{1}{C}_{1}+{K}_{2}{C}_{2}+{K}_{3}{C}_{3}+{K}_{4}{C}_{4})$$

where *K*_1_, *K*_2_, *K*_3_ and *K*_4_ are the calibration coefficients between force and capacitance of each sensor. The shear force along the *x* axis is

3 $${F}_{x}=|{K}_{1}{C}_{1}-{K}_{3}{C}_{3}|$$

while the shear force along the *y* axis is

4 $${F}_{y}=|{K}_{2}{C}_{2}-{K}_{4}{C}_{4}|$$

### Performance of the paper-based supercapacitive sensors

**Figure 6:**
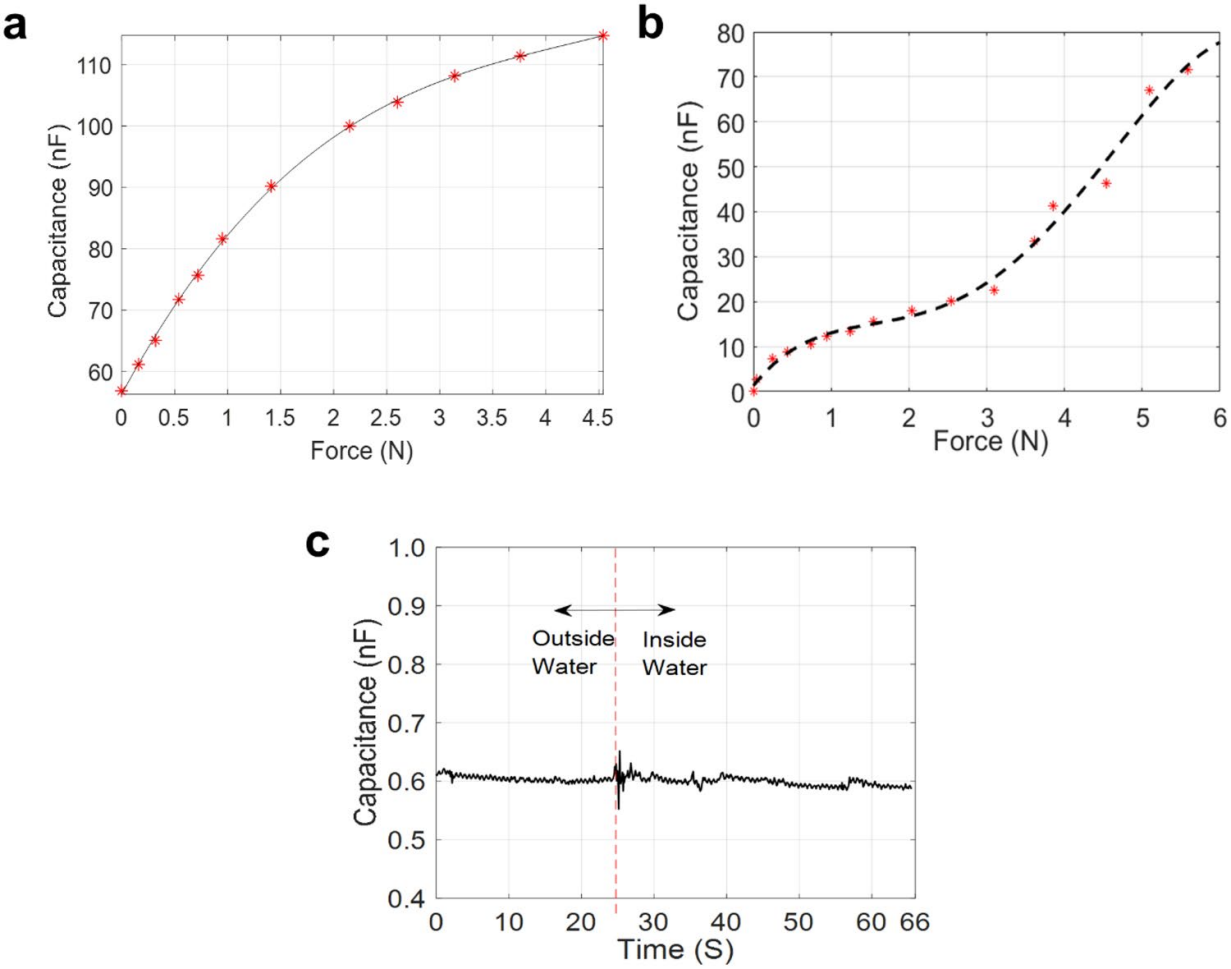
Sensor performance. (**a**) Force response curve of the corrugated electrolyte sensor with an ultra-high sensitivity of 20 nF/N. (**b**) Force response curve of the cloth electrolyte sensor with an ultra-high sensitivity of 20 nF/N. (**c**) Force response curve of a paper-based supercapacitive sensor when immersed into water. The parasitic capacitance in a liquid environment is negligible.

The sensitivity of our paper-based supercapacitive sensors depends on the size and configuration of the sensors, ranging from several nF/N to several μF/N. In contrast, typical sensitivities of traditional MEMS capacitive sensors are on the order of pF/N^50,51^. Figure 6a shows the capacitive response to applied forces for the supercapacitive sensor made of the corrugated electrolyte (Fig. 4a). The sensitivity of the sensor is approximately 20 nF/N. A similar sensitivity can also be obtained for the cloth-based sensor (Fig. 6b). The 20 nF/N sensitivity of the paper-based supercapacitive sensors is more than 1000 times higher than that of traditional MEMS capacitive sensors^50,51^. The range of measurement of the sensors depends on the design of the sensors. The paper-based sensor of Fig. 4c can measure up to a maximum force of around 222.4 N (50 lbs).

In biomedical *in vivo* applications using traditional capacitive sensors, the interference from human tissues on the fringe electric field of the sensors can cause large and highly varying parasitic noise, which contaminates the sensor signal and cannot be eliminated by pre-calibration and subtraction. Our supercapacitive sensors developed do not suffer from this problem. A water-proofed embodiment of the supercapacitive sensor is dipped inside water (Fig. S5, Supporting Information). The force response of the process is shown in Fig. 6c. The capacitance increases by a small amount ranging from 1 pF–10 pF, starting from a base capacitance of 600 pF, which includes variations from instrument errors. This increase in capacitance in the presence of water is negligible compared to the ultra-high sensitivity of the sensor at ~20 nF/N. The parasitic capacitance generally exists in all traditional capacitive sensors and pollutes measured signals during *in vivo* applications. In traditional MEMS electrostatic capacitive sensors, the distance between the electrodes is not negligible relative to the lateral dimensions. The large gap between the electrodes results in additional radial components in the electric field, the so-called fringe electric field^52,53^, which in turn generates parasitic capacitance on the sides of a capacitor. In typical biomedical *in vivo* applications, the magnitude of parasitic noise can be of the order of pF, similar to the sensitivity of MEMS sensor. Supercapacitive sensors do not suffer from parasitic noise. In a supercapacitor, the distance between the positive and negative charges at each electrode is of the order of the size of one or two atomic layers. Hence, the fringe fields are negligible. The measurement characteristics of the new supercapacitive sensors are summarized in Table 1.

**Table 1:** Measurement characteristics of the new supercapacitive sensor.

Sensor sensitivity	Sensor range	Parasitic capacitance	Maximum elongation of sensor electrolyte	Elasticity of sensor electrolyte (Young’s Modulus)	Ultimate tensile strength of sensor electrolyte	Sensor electrolyte shapes
20nF/N	50lbs	<1%	31%	4.5 MPa	1.25 MPa	Arch, cylinder, ring, dome, spiral, corrugated

In conclusion, our study presented a new method for fabricating solid-state electrolytes based on paper substrates coated with ionic gel. Compared to other solid-state electrolytes, the advantages of this paper-based electrolyte are the increased flexibility and the ability to utilize the paper substrate to obtain many different complex geometries for the electrolyte. The ease of making paper substrates of different shapes allows us to construct electrolytes of various complicated 3D geometries. Paper eventually dissolves in the ionic gel, leaving behind a soft highly flexible electrolyte with micro-wrinkles, essential for ensuring high deformability and high sensitivity of the supercapacitive force sensors. The new fabrication method overcomes the several disadvantages of the original ionic-gel electrolytes and of liquid electrolytes, which have seriously limited broad applications of supercapacitive sensors. The sensitivity and range of our paper-based supercapacitive sensors far exceed those of traditional MEMS capacitive sensors. In spite of their superior performance, the new sensors can be fabricated quickly and highly inexpensively with no need of any cleanroom facilities. The ease of fabrication and the versatility of the sensor configurations was demonstrated by a number of force sensors that can measure normal and shear forces. Our method for fabricating paper-based supercapacitive force sensors can be easily customized to specific industrial and biomedical applications and broadly applied to make high-sensitivity force sensors in resource-limited contexts.

## Methods

### Paper-based electrolyte preparation

An IL,1-ethyl-3-methylimidazolium tricyanomethanide [EMIM][TCM] (IOLITEC Inc.), a prepolymer solution, consisting of PEG diacrylate (PEGDA, Mw = 575 g mol^−1^) monomers (Sigma–Aldrich) and a photo initiator of 2-hydroxy-2-methylpropiophenone (HOMPP, Sigma–Aldrich) are mixed in the ratio of 5:4:1 by weight by sonicating. The mixed gel is then brushed on to the filter paper (MF-Millipore, HATF, 0.45 um). The filter paper can be pre-shaped to achieve different geometries. After 1 min under UV exposure, the flexible solid electrolyte is obtained. For comparison with the new electrolyte, ionic-gel electrolytes without the filter-paper ingredient are fabricated by drop-casting the gel (with the same ratio of the components) on to a mold, covering the mold with a piece of glass slide, and exposing under UV light for 1 min.

### Measurement Setup

The capacitance measurements were obtained using a multimeter (DM 3068, Rigol). The force changes on the sensors were recorded using a force gauge (FC50, Torbal, 50 N × 0.01 N). The force gauge was installed on a two-axis linear translational stage (M-406, Newport) with two Vernier micrometers (SM-13, Newport) on each axis. The forces were applied on the sensors by turning the Vernier micrometer. At the same time, the capacitance at each applied force was recorded.

## Electronic supplementary material

Supplementary Document

## Data Availability

The data and results used in all of the figures in this paper will be made available through a public repository at the University of Minnesota.
