# Supplementary material for: Paper-Based Supercapacitive Mechanical Sensors
Source: Sci Rep. 2018 Nov 2;8:16284. doi: 10.1038/s41598-018-34606-1 (PMC6214964; doi:10.1038/s41598-018-34606-1)
Supplement: Supplementary file 1 — Supplementary Document [file 41598_2018_34606_MOESM1_ESM.pdf]

Paper-Based Supercapacitive Mechanical Sensors

Ye Zhang[1], **Serdar Sezen**[2], **Mahdi Ahmadi**[1], **Xiang Cheng**[3] and **Rajesh Rajamani**[1*]

[1]Affiliation, Department of Mechanical Engineering, University of Minnesota, 111 Church St. SE, Minneapolis, MN 55455, USA
[2]Affiliation, Department of Mechanical and Manufacturing Engineering, St. Cloud State University, 720 Fourth Avenue South, Saint Cloud, MN 56301, USA
[3]Affiliation, Department of Chemical Engineering and Materials Science, University of Minnesota, 421 Washington Ave. SE, Minneapolis, MN 55455, USA
E-mail: rajamani@umn.edu

Supporting Information

Paper-Based Supercapacitive Mechanical Sensors

Depth-Varying Images of Paper Dissolution in Ionic Gel

In order to further understand what happens inside the filter paper after the ionic gel is brushed onto it, the ionic gel was diluted using water (IL: PEG: PI: $H2O$ = 5:4:1:20). The diluted mixture was brushed onto the filter paper. Different from the undiluted mixture being brushed onto the filter paper, the filter paper does not turn transparent immediately. Instead, a light white colored film is obtained. This film was exposed under UV light for 1 min, and then placed on a hot plate (60 °C) for 10mins to evaporate the water inside. A small piece from the film is cut and the cross-section is observed under SEM.

The following figure shows an SEM image of the cross-section of the film. From Fig. S1a, it can be seen that the density of the structure changes gradually. That's because the mixture was brushed on the top side of the filter paper, and partially percolated to the bottom side. As a result, the extent of dissolution of the filter paper in the ionic gel varies from high to low. The section near the bottom side (shown in Fig. S1c) kept the porous structure of filter paper, while near the top side (Fig. S1b), the network of the filter paper has been filled with the crosslinked polymer with ionic liquid inside. Later, the fibers in the filter paper will dissolve and wrinkles will be created in the cross-section.

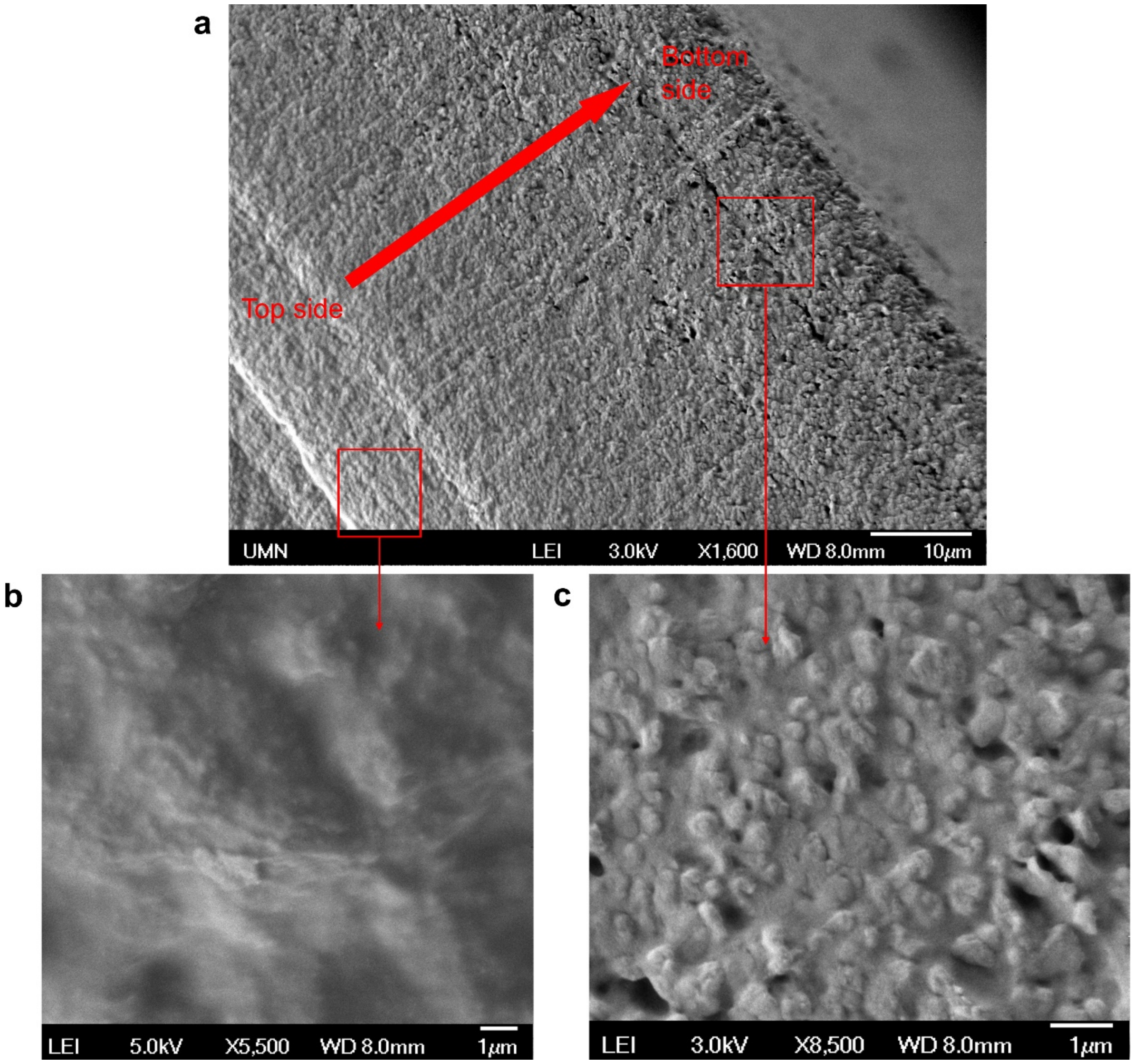

Figure S1. SEM images of the paper- based electrolyte film made using diluted ionic gel. **(a)** depth-varying cross-section of the paper-based electrolyte made by paper being dissolved in diluted ionic gel. (**b)** zoom-in view of the cross-section near the top side. **(c)** zoom-in view of the cross-section near the bottom side.

Ionic Conductivity

Impedance spectroscopy data was collected as real (Z') and imagery (Z") components of the complex impedance by scanning from 10Hz to 1MHz (Autolab). The impedance raw data plot is shown in Fig.S2. The ionic conductivity of the polymer electrolyte film can be determined from the plot by fitting to a simple equivalent circuit shown in Fig. S3[1]. The bulk electrolyte

polarization and resistance to ion motion are represented as the parallel combination of a constant phase element (CPE), which captures the dielectric relaxation and distribution within the bulk polymer electrolyte, and resistance (R_b) respectively. C_i describes the interface polarization and double layer formation, while R_b is the lead resistance. In the impedance plot, R_b and CPE are represented as a depressed semicircle at high frequency, with R_b being the diameter. Nonlinear least squares fitting method was used to estimate R_b at high frequency. The fitted semicircle is plot in red dashed line in Fig. S2. After that, the ionic conductivity (σ) can be obtained using the following equation:

$$\sigma = \frac{L}{R_b A}$$

where L is the traveling length of the ions, A is the area of the electrolyte cross-section. The electrolyte film was transferred on to two Au electrodes (2mm×1mm) with a gap of 0.5mm. The ion conductivity is estimated to be $3\times10^{-4} S/cm$, which enables us to achieve a performance level close to that of the liquid electrolyte-base device.[2]

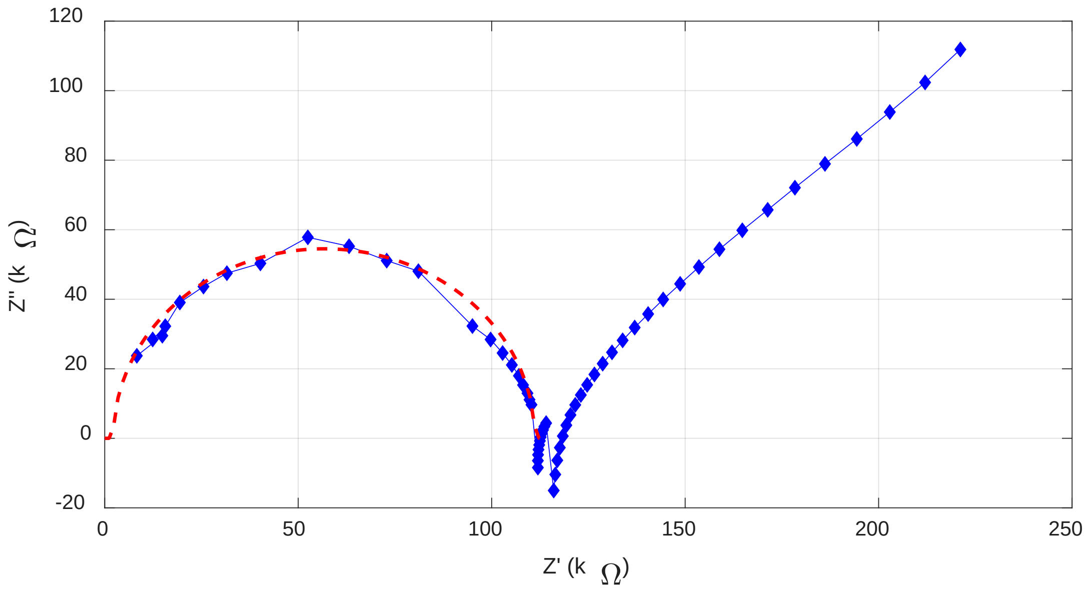

Figure S2. Complex impedance plot

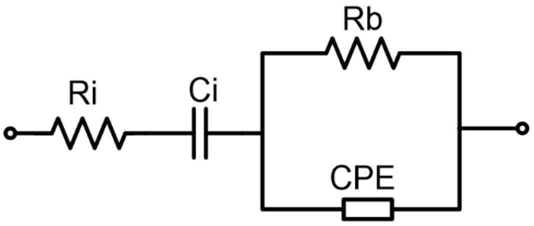

Figure S3. Equivalent circuit of testing cell

Additional Sensor Embodiments

Embodiment S1: Paper-based sensor with an arc shaped electrolyte

Fig. S4a shows a prototype using the new flexible electrolyte fabricated into an electrolyte arch. As force is applied on the top substrate, the electrolyte (arch shaped) comes into contact with the electrolyte at the bottom (Fig. S4b). Increased force results in more contact area between the electrolyte and the electrodes, resulting in higher capacitance. The sensor is made simply by putting two pieces of paper together with double sided tape acting as both the spacing layer and glue as well; the filter-paper based electrolyte is made into an arch shape and placed on top of two parallel copper electrodes.

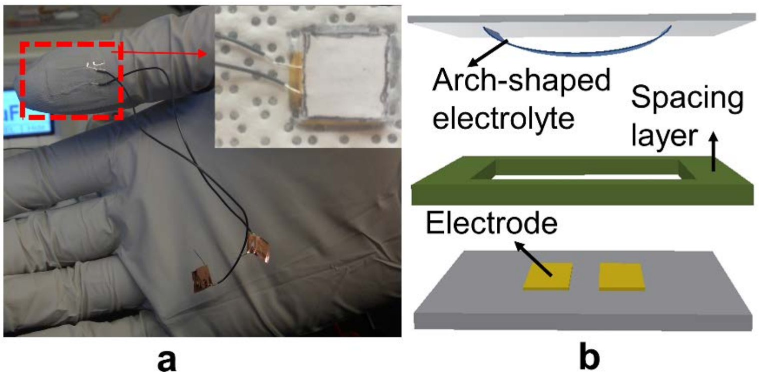

Figure S4. Embodiment of paper-based supercapacitive force sensor. **(a)** photo of the sensor. **(b)** schematic of the sensor with arch-shaped electrolyte

Embodiment S2: Gloves with supercapacitive sensors

Fig. S5a shows a glove with 3 supercapacitive sensors. While grabbing things (inset of Fig. S5a), the force on the top of the sensors will change, which will be translated into a capacitance change. The schematic of the sensors is shown in Fig. S5b. The filter-paper based solid electrolyte is attached to a flexible 3D printed membrane, which is then assembled over the two electrodes. The sensing area of the glove sensor is 4 mm in diameter and the thickness of the sensor is around 1mm.

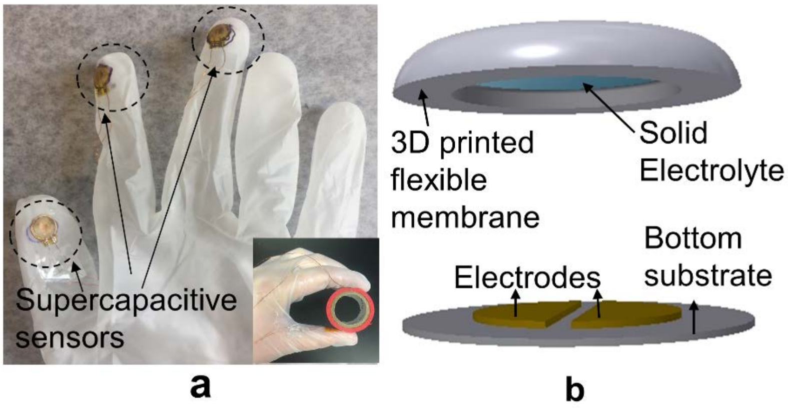

Figure S5. Embodiment of paper-based supercapacitive force sensor used on a glove. **(a)** Photograph. (**b**) Schematic of the glove with the supercapacitive sensors.

References

1. DeLongchamp, D. M. & Hammond, P. T. Fast ion conduction in layer-by-layer polymer films. *Chem. Mater.* **15,** 1165–1173 (2003).
2. Agrawal, R. C. & Pandey, G. P. Solid polymer electrolytes: Materials designing and all-solid-state battery applications: An overview. *J. Phys. D. Appl. Phys.* **41,** (2008)
